# Resonant Subwavelength and Nano-Scale Grating Structures for Biosensing Application: A Comparative Study

**DOI:** 10.3390/s21134523

**Published:** 2021-07-01

**Authors:** Mohammad Abutoama, Marwan Abuleil, Ibrahim Abdulhalim

**Affiliations:** Department of Electrooptics and Photonics Engineering and the Ilse Katz Institute for Nanoscale Science and Technology, School of Electrical and Computer Engineering, Ben Gurion University, Beer Sheva 84105, Israel; abuleil@post.bgu.ac.il

**Keywords:** subwavelength gratings, nano-scale, resonant structures, plasmonics, photonics, multimodal sensing, self-referenced sensing, sef, sers, phase detection, off-the-shelf sensors

## Abstract

Resonant-based sensors are attractive optical structures due to the easy detection of shifts in the resonance location in response to variations in the analyte refractive index (RI) in comparison to non-resonant-based sensors. In particular, due to the rapid progress of nanostructures fabrication methods, the manufacturing of subwavelength and nano-scale gratings in a large area and at a low cost has become possible. A comparative study is presented involving analysis and experimental work on several subwavelength and nanograting structures, highlighting their nano-scale features’ high potential in biosensing applications, namely: (i) Thin dielectric grating on top of thin metal film (TDGTMF), which can support the excitation of extended surface plasmons (ESPs), guided mode resonance, or leaky mode; (ii) reflecting grating for conventional ESP resonance (ESPR) and cavity modes (CMs) excitation; (iii) thick dielectric resonant subwavelength grating exhibiting guided mode resonance (GMR) without a waveguide layer. Among the unique features, we highlight the following: (a) Self-referenced operation obtained using the TDGTMF geometry; (b) multimodal operation, including ESPR, CMs, and surface-enhanced spectroscopy using reflecting nanograting; (c) phase detection as a more sensitive approach in all cases, except the case of reflecting grating where phase detection is less sensitive than intensity or wavelength detection. Additionally, intensity and phase detection modes were experimentally demonstrated using off-the-shelf grating-based optical compact discs as a low-cost sensors available for use in a large area. Several flexible designs are proposed for sensing in the visible and infrared spectral ranges based on the mentioned geometries. In addition, enhanced penetration depth is also proposed for sensing large entities such as cells and bacteria using the TDGTMF geometry.

## 1. Introduction

Resonant-based optical structures exhibit clear and well-defined features which can be efficiently used in a wide range of applications, such as sensing [1,2,3,4]. In particular, resonant dips or peaks in the reflection, transmission, or absorption spectra of the resonant structures are sensitive to variations in the analyte refractive index (RI) of the material surrounding the structure surface. Monitoring variations in the material properties with high sensitivity, easy detection, high precision, reliability, low cost, and fast response is important in biology, chemistry [3,4], medicine, the environment, and industry. With the rising nanostructures fabrication methods, the manufacturing of subwavelength and nano-scale gratings (nanogratings) in a large area and at a low cost has become possible and even controllable. Nanogratings are planar and easy to use compared to structures requiring prism, fiber, or waveguide coupling. The periodic structure is homogeneous over a much larger scale than the period. Therefore, there are no problems or variability over the sensor area as compared to other types of sensing substrates, such as nanoparticles spun or sputtered on the surface. These sensing substrates usually have random distribution but their randomness varies, causing variability of the measured signal.

When referring to nanogratings, researchers usually mean gratings having nano-scale features such as grating thickness (h), period (Λ), and/or line (w)/space (a) width less than 400 nm. Subwavelength gratings (SWGs), on the other hand, refer to gratings with Λ smaller than the wavelength (λ) in which diffraction does not occur, meaning that any change (dip, peak) in the spectra is mainly attributed to optical resonant mode excitation and not to diffraction. In many cases, the word ‘nano’ refers to the period being at the nanoscale without much limitation on the height. The enhanced interaction between the analyte and the electromagnetic field (EMF) exists near the surface. The flexibility in designing the resonant conditions makes the resonant SWG and nanograting-based structures one of the most important optical structures for use in different sensing modes such as yes/no type sensors, RI sensing, surface-enhanced fluorescence (SEF), and surface-enhanced Raman scattering (SERS) sensing.

Several resonant mechanisms can be supported by plasmonic/photonic substrates and utilized to design SWG and nanograting-based sensors: (i) Metallic nanograting on metallic substrate or on thin metal film (MF) on dielectric substrate for conventional extended surface plasmon resonance (ESPR) and cavity modes (CMs) excitation [5,6,7,8,9]; (ii) thin metallic nanoslits on dielectric substrate for enhanced optical transmission (EOT) resonance excitation [10,11,12,13]; (iii) thin dielectric grating on top of thin MF (TDGTMF) or on bulk metal, which can support the excitation of extended plasmons, guided mode resonance (GMR), or leaky mode [14,15]. The authors of [16,17] used a thin MF combined with thick dielectric SWG (>400 nm); (iiii) Thin dielectric grating coupled to the waveguide layer on dielectric substrate for GMR excitation [18,19]; (iv) thick dielectric SWG exhibiting GMR without a waveguide layer [20].

In this work, we provide a brief theoretical background on the grating coupling geometry with an example of coupling the incident photons to the ESPs in the case of shallow metallic gratings. Then, the analysis of three SWG/nanograting-based sensors is presented as proposed by our group, highlighting their relevance in sensing applications.

A new technique for designing a self-referenced biosensor in the grating coupling geometry based on the combination of a thin dielectric grating with a thin MF was proposed in the first nanograting configuration [21,22]. This strategy makes the measurement stable and less sensitive to temperature fluctuations and optomechanical drifts. Multiple resonant modes of reflecting nanograting are utilized in the second nanograting configuration for proposing and experimentally demonstrating a simple multimodal sensing operation, including ESPR, SEF, and SERS [23]. This is important for simultaneously achieving as much information as possible on the analyzed sample. Resonant modes are theoretically and experimentally demonstrated in thick enough subwavelength dielectric grating without using the planar waveguide layer in the third grating configuration [20].

Finally, new experimental results on the implementation of intensity and phase detection as a sensitive detection method based on off-the-shelf nanograting sensors are presented. The prepared samples were shown to be easily and reproducibly extracted from optical compact discs (CDs) without the need any chemical or other treatment on the extracted pieces. Despite the progress made in nanofabrication methods, manufacturing of high-quality gratings at a low cost and in a large area is still challenging and requires high-quality equipment. Recently, many works have reported the use of off-the-shelf devices for a wide range of applications. Particularly, optical devices supporting plasmon’s excitation, such as CDs, are of high interest for practical applications. CD-recordable (CD-R), digital versatile disc (DVD), and Blu-ray disc (BD) are well-known types of commercial optical discs used for data storage, and they usually consist of polycarbonate, data-storage material (such as dye film), and metallic films (mostly made of silver, gold, or aluminum). These structures are available and can be easily implemented in several applications, including sensing, with a low cost and rapid and easy detection. These structures can also be used in a large sensing area.

In all the configurations below, we highlight the nano-scale features of each geometry and their use in sensing applications. Among the unique features of the proposed SWG/nanograting structures, we highlight the following: (a) The flexibility to design the sensors for sensing small (molecules, viruses) and large (cells) entities by utilizing the excited long-range SPR (LRSPR) and engineering the penetration depth of the evanescent wave to be large enough [21]; (b) the flexibility of sensing different types of analytes; (c) the possibility of working at several operation modes: Spectral, angular, intensity, phase detection, and intensity; (d) the flexibility of tuning the operation spectral range (visible, infrared (IR)), (e) planar and compact configurations, which are preferable over prism-based structures in the sense of miniaturization and implantation in integrated photonics and plasmonics. All the reflection spectra and phase calculation for all configurations were performed using COMSOL Multiphysics.

### 1.1. ESP in the Grating Coupling Geometry

An important technique of coupling the light falling on the optical structure to their optical modes is the grating coupling geometry, in which the additional momentum is provided by the grating itself. To understand the role of the grating in supporting the optical mode excitation in the structure, we consider ESP excitation in shallow metallic grating as an example by deriving the ESP dispersion relation. The ESP is characterized as a longitudinal charge density distribution generated at the interface between metal and dielectric materials and excited under transvers magnetic (TM) polarized light having the well-known dispersion relation given in Equation (1):

(1) $$k_{SP}=\left(\omega /c\right)\sqrt{\varepsilon_{m}\varepsilon_{d}/\left(\varepsilon_{m}+\varepsilon_{d}\right)}$$

where $k_{SP}$ is the k-vector of the SP and $\varepsilon_{m}$, $\varepsilon_{d}$ are the dielectric constants of the metal and dielectric materials, respectively. ω is the light frequency, and c is the light velocity in vacuum.

Considering the case of grating where the dielectric material (analyte medium) exists within the grating spaces and on top of the grating (superstrate), the equation for calculating the spectral position of the resonance is called the plasmon momentum matching equation. The plasmon momentum matching equation is given by $k_{xi}+k_{G}=\pm k_{SP}$, where $k_{xi}$ is the k-vector of the incident light in the polarization direction (*x*-axis), $k_{G}$ is the k-vector of the grating required to provide the additional momentum $k_{G}=\left(2\pi /\Lambda \right)m\;$to excite the ESP, and $k_{SP}$ is the k-vector of the ESP as expressed in Equation (1). The plasmon momentum matching equation can be written in the following way [24]:

(2) $$k_{0}n_{d}sin\theta +\left(\frac{2\pi }{\Lambda }\right)m=\pm k_{0}\sqrt{\frac{\varepsilon_{m}\varepsilon_{d}}{\varepsilon_{m}+\varepsilon_{d}}}$$

where *n_d_* is the dielectric medium RI and *θ* is the incident angle, *m* is the diffraction order (integer), the sign ‘+’ in the right side of Equation (2) corresponds to (*m* > 0), and the sign ‘−’ corresponds to (*m* < 0). At normal incidence (*θ* = 0°), the excitation wavelength of the ESP can be calculated using Equation (3):

(3) $$\lambda_{exc}=\frac{\Lambda }{m}\sqrt{\frac{\varepsilon_{m}\varepsilon_{d}}{\varepsilon_{m}+\varepsilon_{d}}}$$

As arises from Equation (3), at normal incidence, only the momentum supported by the grating (2πm/Λ) can excite the ESP. Hence, the condition |m|>0 should be satisfied. In addition, at normal incidence, the excitation wavelength for the positive and negative diffraction orders is equal. For the sake of simplicity, the simple Drude model for the dielectric function of the metal $\varepsilon_{m}=1-\left(\omega_{p}{}^{2}/\omega^{2}\right)$ ($\omega_{p}$ is the plasma frequency) is substituted into Equation (2). Then, after ordering the equation, the following relation is achieved:

(4) $$\omega^{2}=\frac{1}{2}\left[\omega_{p}{}^{2}+{\left(k_{xi}+k_{G}\right)}^{2}c^{2}\left(1+\frac{1}{\varepsilon_{d}}\right)-\sqrt{\omega_{p}{}^{4}+2{\left(k_{xi}+k_{G}\right)}^{2}c^{2}\omega_{p}{}^{2}\left(1-\frac{1}{\varepsilon_{d}}\right)+{\left(k_{xi}+k_{G}\right)}^{4}c^{4}{\left(1+\frac{1}{\varepsilon_{d}}\right)}^{2}}\right]$$

Dividing Equation (4) by $\omega_{p}{}^{2}$ and defining the normalized parameters (the plasma frequency, $\omega_{n}=\omega /\omega_{p}$, and the plasmon wave-vector, $k_{n}=k_{SP}/k_{p}=\left(k_{xi}+k_{G}\right)/k_{p}$ where $k_{p}=\omega_{p}/c$), the dispersion relation for the ESP excitation in the case of the grating coupling can be presented in the normalized form in a similar way to the prism coupling case:

(5) $$\omega_{n}{}^{2}=\frac{1}{2}\left[1+k_{n}{}^{2}\left(1+\frac{1}{\varepsilon_{d}}\right)-\sqrt{1+2k_{n}{}^{2}\left(1-\frac{1}{\varepsilon_{d}}\right)+k_{n}{}^{4}{\left(1+\frac{1}{\varepsilon_{d}}\right)}^{2}}\right]$$

**Figure 1:**
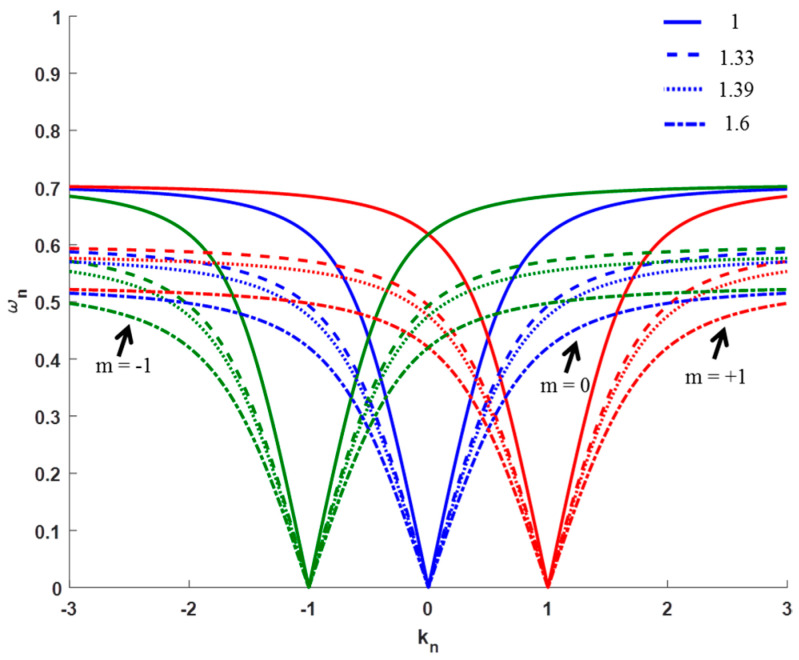
Dispersion relation for ESPM in the grating coupling geometry at different diffraction orders and analyte RIs.

Figure 1 shows the dispersion relation for the case of the grating coupling for three different diffraction orders (m = −1, 0, 1) as an example. Since sensing applications are the topic of this work, the ESP dispersion relation in the grating geometry was plotted for four RIs of analytes to show the variation in the plasmon excitation condition as the analyte changes. The solid curves in Figure 1 are for air (*nd* = 1), the dashed curves are for *nd* = 1.33 (close to water), the dotted curves are for *nd* = 1.39 (close to glycerol), and the dashed-dotted curves are for *nd* = 1.6 (close to other organic materials). This demonstrates the fact that the ESP in the grating geometry can be used for sensing of different types of analytes such as gases, water, and organic materials. Assuming fixed normalized momentum *kn,* the normalized frequency *ωn* decreases as the anlyte RI increases, as can be observed from Figure 1. Note that the sensitivity becomes larger as *k_n_* becomes further from the exact matching condition.

### 1.2. ESP Propagation Length and Penetration Depth

The fact that the ESP wave has a propagating field in the x direction and a decaying field in the z direction make it a potential candidate to be used in integrated photonics and sensing applications, respectively. One of the serious problems that the ESP suffers from is the limited penetration depth, which limits its use as a sensor for only small entities existing near the surface. Hence, special techniques for enhancing the penetration depth of the ESP wave are of high interest for sensing large entities, such as cells and bacteria, which is not possible using the conventional ESP geometry. One option is the use of LRSP excited in the grating coupling configurations [13,21], which is shown later. Recent works have reported progress in overcoming the penetration depth limitation [25,26]. The propagation length of the ESP wave depends on the imaginary part of the propagation constant $k_{x}$ in Equation (1) [27]. The real ($k_{xr}$) and imaginary parts ($k_{xi}$) of $k_{x}$ are expressed as:

(6) $$k_{xr}=\left(\omega /c\right)\sqrt{\frac{\varepsilon_{mr}\varepsilon_{d}}{\varepsilon_{mr}+\varepsilon_{d}}};\;k_{xi}=\left(\omega /c\right){\left(\frac{\varepsilon_{mr}\varepsilon_{d}}{\varepsilon_{mr}+\varepsilon_{d}}\right)}^{3/2}\left(\frac{\varepsilon_{mi}}{2\varepsilon_{mr}{}^{2}}\right)$$

where $\varepsilon_{mr}$ and $\varepsilon_{mi}$ are the real and imaginary parts of $\varepsilon_{m}$ respectively. The ESP wave propagation length ($L_{x}$) along the metal surface is expressed as:

(7) $$L_{x}=\frac{1}{2\left|k_{xi}\right|}=\left(\frac{\lambda }{2\pi }\right){\left(\frac{\varepsilon_{mr}+\varepsilon_{d}}{\varepsilon_{mr}\varepsilon_{d}}\right)}^{3/2}\left(\frac{\varepsilon_{mr}{}^{2}}{\varepsilon_{mi}}\right)$$

As can be concluded from Equation (7), $L_{x}$ is limited by the imaginary part of the dielectric function of the MF ($\varepsilon_{mi}$). Otherwise, the SP wave might theoretically propagate to infinity.

On the other hand, the ESP penetration depth (δ) inside the metal ($\delta_{m}$) and the dielectric ($\delta_{d}$) are given by Equation (8):

(8) $$\delta_{m}=\left(\frac{\lambda }{2\pi }\right)\sqrt{\frac{\varepsilon_{mr}+\varepsilon_{d}}{-\varepsilon_{mr}{}^{2}}};\;\delta_{d}=\left(\frac{\lambda }{2\pi }\right)\sqrt{\frac{\varepsilon_{mr}+\varepsilon_{d}}{-\varepsilon_{d}{}^{2}}}$$

**Figure 2:**
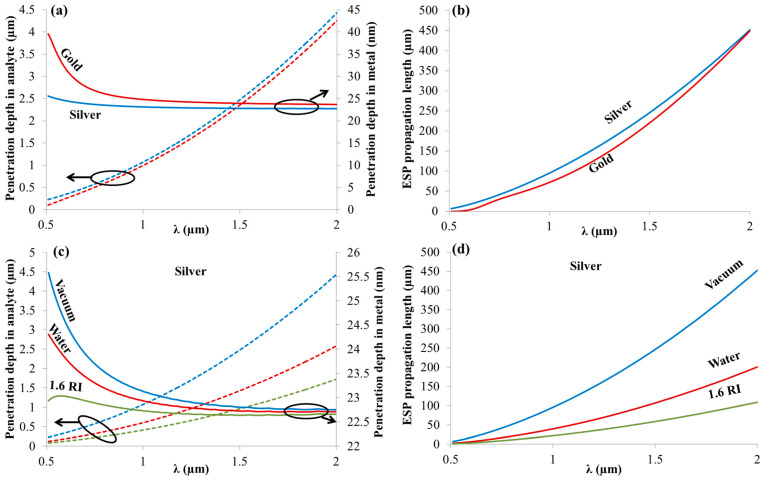
(**a**) Penetration depths of an ESP wave in vacuum (dashed curves) and in metal (solid curves) for silver (blue) and gold (red); (**b**) ESP propagation lengths along silver/vacuum (blue) and gold/vacuum (red) interfaces; (**c**) penetration depths of an ESP wave in metal (solid curves) for silver and for different types of analyte mediums: Vacuum (blue), water (red), and 1.6 RI close to organic material (green); (**d**) ESP propagation lengths along silver/analyte interface for different types of analytes: Vacuum (blue), water (red), and (1.6 RI). The dispersion of water was considered in the calculations.

Figure 2a shows the penetration depths of an ESP wave in vacuum (dashed curves) and in metal (solid curves) for silver (blue) and gold (red). As observed from Figure 2a, the penetration depth in analyte (*δd*) is slightly larger for silver than for gold, but the values are very comparable. However, the penetration depth in metal (*δm*) is larger for gold than for silver. This means more absorption will occur in case of gold than in silver, resulting in lower quality factor of the resonance. Additionally, *δd* is much larger in the near IR (NIR) than in the visible wavelengths due to the larger ratio between the real and imaginary parts of the dielectric function as the wavelength increases. On the other hand, *δm* is larger for the visible wavelengths than the NIR ones, as expected. Figure 2b shows the ESP propagation lengths (*Lx*) along silver/vacuum (blue) and gold/vacuum (red) interfaces where a larger propagation length is seen for silver in the given wavelength range. Figure 2c shows δd (dashed curves) and *δm* (solid curves) for silver and for different types of analytes: Vacuum (blue), water (red), and (1.6 RI close to organic material). In the whole spectral range, δd and δm decrease when the analyte RI increases. Figure 2d shows *Lx* along silver/analyte interface for different types of analyte mediums: vacuum (blue), water (red), and (1.6 RI), where *Lx* decreases when the analyte RI increases for the whole spectral range. As can be seen, there is an inverse correlation between the penetration depth in the dielectric and the propagation length because the increase in the penetration depth means that most of the electromagnetic energy is in the dielectric. Therefore, there is less dissipation in the metal, and the wave can propagate more along the interface. As a final remark, for sensing operation, silver has an advantage over gold with respect to its larger penetration depth and propagation length. On the other hand, silver can be easily oxidized, and a protection method is required.

## 2. SWG and Nanograting-based Sensors Configurations

### 2.1. Thin Dielectric Grating on Thin MF (TDGTMF)-based Self-Referenced Sensor

**Figure 3:**
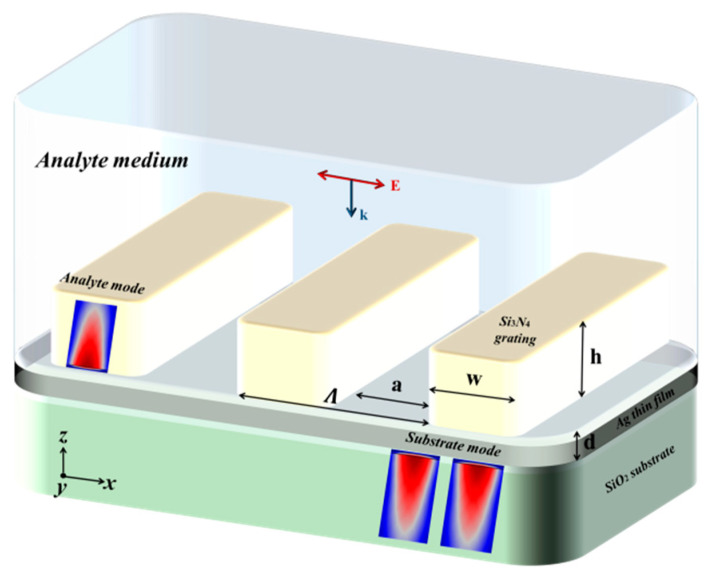
Schematic diagram of the self-referenced sensor based on TDGTMF geometry.

A new technique for designing a self-referenced biosensor in the grating coupling geometry based on the combination of a thin dielectric grating (<200 nm) with a thin MF (<50 nm) is proposed in Figure 3, which has been shown to exhibit the excitation of two optical modes [21,22]. The first optical mode is excited as a result of exciting the GMR supported by the dielectric grating, showing a reflection dip instead of peak due to the existence of the MF below the dielectric grating. Since the EMF associated with the excitation of the first mode, it exists mostly in the analyte medium side. This mode is called analyte mode. On the other hand, the second optical mode is ESP mode (ESPM), excited at the MF-substrate interface (called the substrate mode).

**Figure 4:**
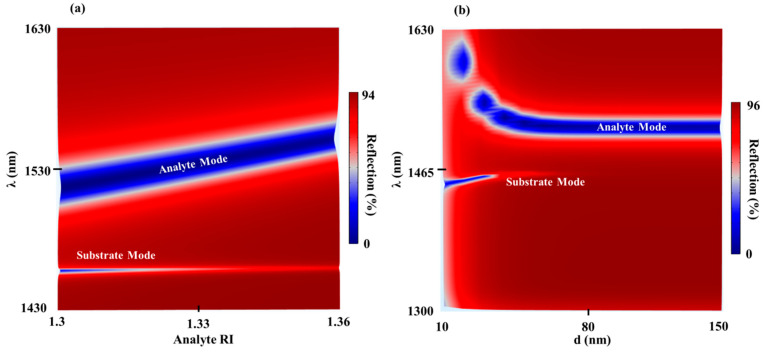
(**a**) Reflection analyte RI–λ map of the TDGTMF geometry for h = 175 nm and d = 40 nm. (**b**) Reflection d–λ map from the TDGTMF geometry, with h = 175 nm. TM polarized light under normal incidence was used in the simulations of the Si3N4 grating with Λ = 1000 nm and FF = 0.55 in (**a**,**b**). Water and SiO_2_ were used for the superstrate (analyte) and substrate, respectively.

Because the substrate mode is not sensitive to the analyte RI, this mode was used as a reference for detecting variations in the analyte RI as shown in the reflection analyte RI–λ map of the TDGTMF geometry for h = 175 nm and d = 40 in Figure 4a. It is interesting to note that, as the increased RI increases, the substrate mode started to disappear. Calculations showed that it almost disappeared at the analyte RI, close to the SiO_2_ substrate RI (1.443–1.445 in the given spectral range). Reflection d–λ map from the TDGTMF geometry, with h = 175 nm, is shown in Figure 4b. The MF thickness was shown to have a critical role in exciting the substrate mode (Figure 4b), where, above a certain value (~80 nm), the field cannot penetrate through the MF and therefore cannot excite the ESP at the lower interface of the MF. Hence, the substrate mode disappears.

**Figure 5:**
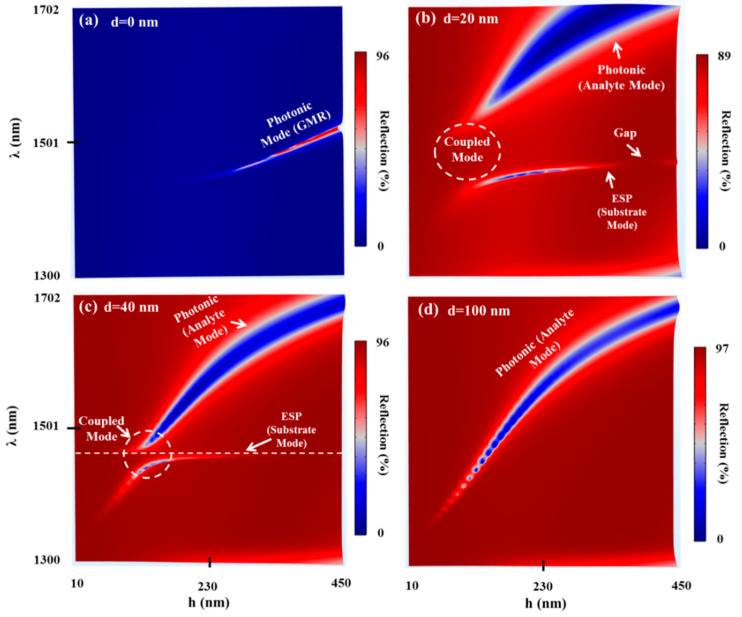
Reflection h–λ maps of the TDGTMF geometry for different values of d: (**a**) d = 0 nm, (**b**) d = 20 nm, (**c**) d = 40 nm, and (**d**) d = 100 nm. TM polarized light under normal incidence was used in the simulations of the Si3N4 grating with Λ = 1000 nm and FF = 0.55. Water and SiO_2_ were used for the superstrate (analyte) and substrate, respectively.

**Figure 6:**
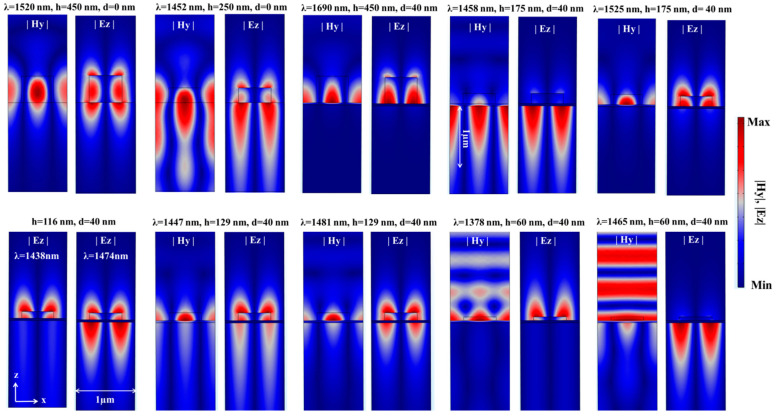
FD at the resonant wavelengths of the TDGTMF geometry at different combinations of h and d. TM-polarized light under normal incidence was used in the simulations of the Si3N4 grating with Λ = 1000 nm and FF = 0.55. Water and SiO_2_ were used for the superstrate (analyte) and substrate, respectively.

**Figure 7:**
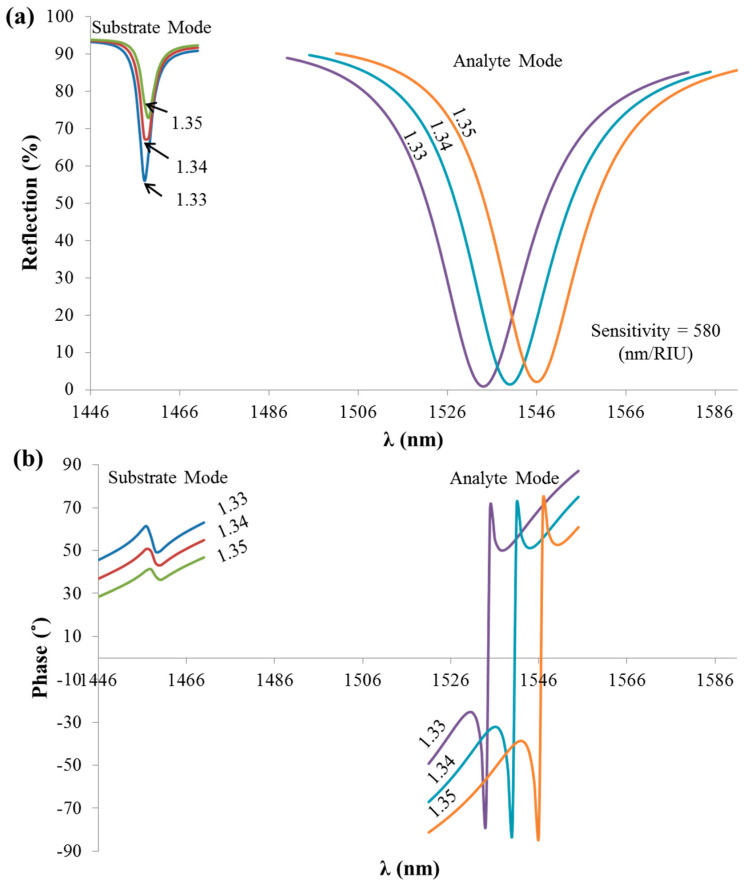
(**a**) Reflection spectra from the TDGTMF geometry at different analyte RIs. (**b**) Phase response at the same RIs as in Figure 7a. TM-polarized light under normal incidence was used in the simulations of the Si3N4 grating, with Λ = 1000 nm and FF = 0.55. SiO_2_ was used for the substrate.

The analyte mode excitation wavelength, on the other hand, did not change when the MF thickness increased above ~ 60 nm, as shown in Figure 4b. TM-polarized light under normal incidence was used in the simulations of the Si3N4 grating with Λ = 1000 nm and FF = 0.55 in Figure 4, Figure 5, Figure 6 and Figure 7. Water and SiO_2_ were used for the superstrate (analyte) and substrate, respectively.

Although the TDGTMF was previously proposed by our group [21,22], there are some questions that still must be answered regarding this geometry. First, we strengthened our understanding of the nature of the resonant modes excited in the structure for different values of the grating parameters and MF thicknesses. New data are presented in Figure 5, which show the reflection h–λ maps of the TDGTMF geometry for different values of d. Without the MF (Figure 5a), the resonant branch is associated with the excitation of pure GMR. For d = 20 nm (Figure 5b) and d = 40 nm (Figure 5c), the TDGTMF geometry supports the excitation of both the photonic (GMR on MF) and plasmonic mode (ESP excited at the MF interface with the SiO_2_ substrate). As can be observed from Figure 5c for the case of d = 40 nm, the photonic branch splits around the horizontal dashed white line, which indicates the ESP wavelength of the substrate mode. The circled area indicates the region in which a strong coupling between the photonic and plasmonic modes is observed, which is associated with the FD of both the GMR on MF and the ESP modes, as in Figure 6. At d = 100 nm in Figure 5d, the ESP at the lower MF surface almost disappears. Therefore, a photonic mode branch is observed again. An important observation is shown at d = 20 nm, in which a gap in the continuous ESP substrate mode branch is observed. The origin of this gap requires more investigation, and we believe that it might be related to the bound state in the continuum (BIC) observed in several optical configurations.

To strengthen our understanding of the nature of the resonant modes in Figure 5, FD calculations (|Hy| and |Ez|) are shown in Figure 6 for at different combinations of h and d.

We started with the pure GMR at h = 450 nm and d = 0 nm. The resonant wavelength was λres = 1520 nm, and |Hy| was well confined within the grating line, since the grating was thick enough to be able to effectively support the GMR, as observed in Figure 5a. On the other hand, at h = 250 nm and d = 0 nm, λres = 1452 nm, |Hy| was less confined within the grating line, and the field was leaky (leaky mode).

When involving the MF and at h = 450 nm and d = 40 nm, λres = 1690 nm, and |Hy| existed within the grating spaces and lines as expected from the GMR on MF geometry. On the other hand, at h = 175 nm and d = 40 nm, the coupling regime was still relatively far (see the circled area in Figure 5c), and two resonant wavelengths were observed in Figure 5c. At λres1 = 1525 nm, |Hy| was mainly concentrated within the grating spaces and lines, while at λres2 = 1458 nm, |Hy| existed almost only within the substrate medium as expected. The case of h = 129 nm and d = 40 nm fell with the coupled modes regime. Therefore, the resonant wavelengths λres1 = 1481 nm and λres1 = 1447 nm showed the signatures of both photonic and plasmonic FD nature. The case h = 116 nm and d = 40 nm was also close to the coupling regime, and therefore, both the photonic and plasmonic FD signature was observed (it was clearer at 1474 nm than at 1438 nm). Finally, at h = 60 nm and d = 40 nm, at λres1 = 1465 nm, the resonant branch returned to ESP branch behavior, as seen from the FD. Similarly, at λres1 = 1378 nm, the resonant branch returned to the photonic branch. The substrate mode has a larger penetration depth than the analyte mode [21,22], indicating long-range SP (LRSP) excitation. Thus, it can be used for large bioentity detection, such as cells and bacteria. To see this, field distribution (FD) for the case of h = 175 nm and d = 40 nm is shown in Figure 6. Field distributions were λ = 1458 nm, indicating the substrate mode resonant wavelength, while λ = 1525 nm indicates the analyte mode resonant wavelength.

Phase detection has the advantage of having sharp response at resonance in comparison to intensity detection. Hence, the resonance wavelength can be easily determined in a high precision, as observed in Figure 7. Figure 7a shows the spectral reflection variation at different analyte RIs (1.33, 1.34, and 1.35) in which the width of the resonance (FWHM) is large, while in Figure 7b, a sharper response was observed when detecting the phase for the same RIs in Figure 7a. The spectral reflection sensitivity of the analyte mode is defined as the ratio between the shift in the resonant wavelength and the shift in the analyte RI, given by ∆λ/∆n = 580 (nm/RIU), as indicated in Figure 7a. As can be observed in Figure 7b, the spectral phase response of the analyte was much sharper than the one associated with the substrate mode. This is expected to be a result of the losses of the metal in the case of the ESP substrate mode, which significantly affects the sharpness of the phase response expected at resonance. Profiting from the sharp jump in phase function under ESPR excitation, an improved detection limit using phase over wavelength detection was demonstrated by the authors of [28]. In particular, the polarimetric approach was applied at the spectral mode, and a clear advantage of the phase over the intensity or wavelength detection was shown both experimentally and theoretically using two different detection limit estimation algorithms. The system noise was also considered in the performance analysis. More details on the mathematical analysis of the detection limit have been presented by the authors of [28].

**Figure 8:**
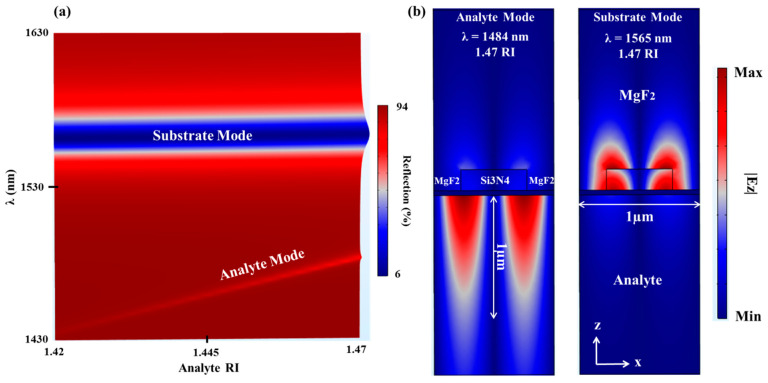
(**a**) Reflection analyte RI–λ map of the TDGTMF geometry for h = 175 nm and d = 40 nm when replacing the analyte medium (ambient or superstrate) with MgF2, while the substrate was replaced with a RI close to that of SiO_2_ (1.42–1.47 RI), for example, with blood or serum as the medium. (**b**) FD at the resonant wavelengths in Figure 8a at 1.47 analyte RI. TM-polarized light under normal incidence was used in the simulations of the Si3N4 grating, with Λ = 1000 nm and FF = 0.55.

The idea behind the proposed self-referenced sensor configuration was stimulated from a work performed by our group. Our previous work showed that showed that, using a thin enough metallic grating (~20–40 nm), the excitation of two EOT peaks is possible [13,29]. The EOT substrate mode was also used as a reference and showed a larger penetration depth than the analyte mode. A clear advantage of the proposed self-referenced sensor, previously described by our group in [21], over the one based on the EOT in described in [13] is the use of a thin dielectric grating with lateral features in the sub-micron scale and a thin MF instead of a thin metallic nanoslits (40–50 nm slits) array, which requires a fine lithography process. An important remark on the LRSP in the substrate medium in the TDGTFM geometry in the regard to sensing applications is that it can also be used for sensing with high sensitivity and large penetration depth, for example, by replacing the analyte medium (ambient) with a medium of low RI such as MgF2. On the other hand, the substrate is replaced with a liquid medium of RI close to that of SiO_2_, for example, with blood or serum as the medium. To demonstrate this, numerical simulation was carried out and shown in Figure 8 below.

In Figure 8, the analyte medium (ambient or superstrate as shown in Figure 3) was replaced with MgF2 (meaning that the Si3N4 grating spaces were filled with MgF2), while the substrate was replaced with an RI close to that of SiO_2_ (1.42–1.47 RI), for example, with blood or serum as the medium. Figure 8a shows reflection analyte RI–λ map of the TDGTMF geometry for h = 175 nm and d = 40 nm, where opposite behavior to that in Figure 4a was observed. The bottom branch is now called the analyte mode branch, while the top one is related to the substrate mode, which almost did not change with the analyte RI. FD at the resonant wavelengths in Figure 8a at 1.47 analyte RI is shown in Figure 8b to verify the claim above, in which larger penetration depth was observed in the analyte medium. TM-polarized light under normal incidence was used in the simulations of the Si3N4 grating, with Λ = 1000 nm and FF = 0.55.

To summarize, this design proposed for water sensing in the infrared (IR) spectral range and the distance between the two dips (associated with the analyte and substrate modes excitation) in the reflection spectra is small. The design can be easily tuned to work in different types of analyte mediums by tuning the nano-scale features of the grating parameters as partially shown above, mainly by varying h. To work at the visible spectral range, the grating period can be reduced accordingly. Another important parameter that can help in the design is the FF.

### 2.2. Reflecting Nanograting-Based Multimodal Sensor

**Figure 9:**
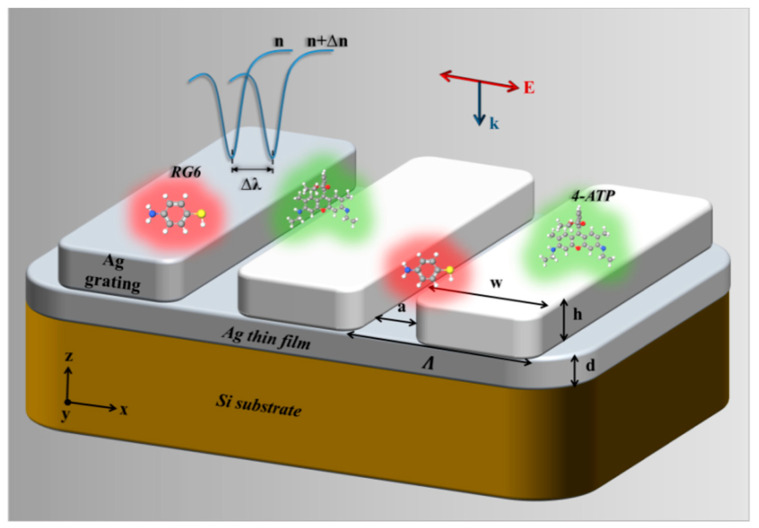
General schematic of the metallic grating on thin MF geometry under free-space excitation (normal incidence and TM polarization) for multimodal sensing. The RG6 and 4-ATP molecules were embedded to demonstrate the potential of the reflecting grating for SEF and SERS applications, respectively. The sensing application of the structure is demonstrated by the blue curves at the top-left part of the figure. Changing the refractive index of the surrounding material from n to n + Δn leads to a change in the wavelength of the minimum of the reflectance spectra.

Metallic nanogratings can be effectively implemented for sensing applications due to the optical mode excitation they can support. The nature of the resonant modes of reflecting gratings (metallic grating on MF, as shown in Figure 9) with nano-grooves was theoretically and experimentally investigated as a function of the λ, h, and Λ under normal incidence and TM polarization [24]. Figure 9 shows the general scheme of the proposed geometry, consisting of a metallic grating on a thin MF of thickness d (>100 nm), which is thick enough to suppress transmission inside the silicon (Si) substrate under free-space excitation. The main conclusions arising from the mentioned work were as follows: For thin enough (20 nm) gratings, the resonant modes at the investigated λ range (500–1400 nm) were mainly attributed to ESPMs excitation. As mentioned in the theoretical background above, the resonant wavelength (assuming fixed incident angle) in the limit of shallow gratings can be simply determined by the RIs of the grating and the surrounding materials as well as by Λ, without much dependence on h [24], and can be estimated using Equation (2) above. Increasing h (at which the plasmon momentum matching equation no longer accurately predicts the SP’s resonance position) allows the excitation of cavity modes (CMs) (excited within the grooves of the grating) that might also couple to ESPs in what we called ESP-cavity hybrid mode. Field calculations were performed expressing the nature of the three-mode field (ESP, CM, and ESP-cavity hybrid mode [24]). Experimental verification (RI sensing in the visible and IR ranges and SEF experiments) of the observed phenomena was performed on a 154 nm-thick silver (Ag) nanograting with 1050 nm period fabricated using electron beam lithography. The whole issue of supporting the CMs is that the grating has very narrow grooves where the slit width is much smaller than the grating period and wavelength, as shown below in detail.

**Figure 10:**
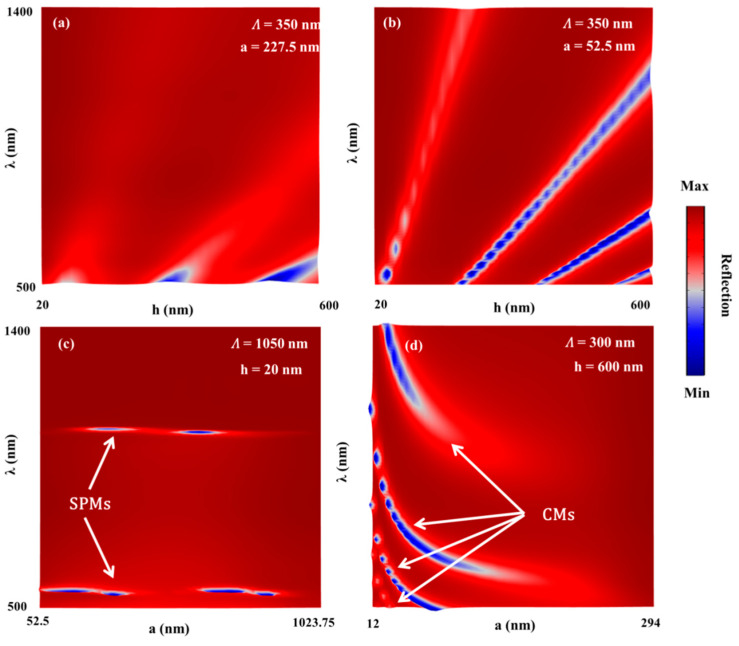
Reflection h-λ maps from the reflecting grating, with Λ = 350 nm. Two cases were simulated: (**a**) a = 227.5 nm, (**b**) a = 52.5 nm. Reflection a-λ maps from the reflecting grating with different combinations of the grating period (Λ) and thickness (h): (**c**) Λ = 1050 nm and h = 20 nm, (**d**) Λ = 300 nm and h = 600 nm. TM-polarized light under normal incidence was used in all simulations of the Ag grating. Air and Si used for the superstrate and substrate respectively.

Since grating with nano-scale features is the topic of this work, the analysis of the reflecting grating at different grating grooves width (a) and thickness (h) at the nano scale is presented in Figure 10. TM-polarized light under normal incidence was used in all simulations of the Ag grating. Air and Si were used for the superstrate and substrate, respectively. The behavior of the reflecting grating as a function of the groove width (a) varied significantly for different values of h and Λ. The reflection h-λ maps from the reflecting grating with Λ = 350 nm are shown in Figure 10 for a = 227.5 nm (Figure 10a) and a = 52.5 nm (Figure 10b). For the narrow grooves in Figure 9, four resonant branches were clearly observed and associated with the CMs excitation. On the other hand, for the wide grooves in Figure 10a, the CMs were observed only for the short wavelengths. The special case in Figure 10a,b with Λ = 350 nm was used to make sure that only CMs could be excited (ESPMs cannot be excited at the given spectral range).

In order to strengthen the importance of the groove width in the reflecting gratings, reflection a-λ maps from the reflecting grating with different combinations of the Λ and h are shown in Figure 10c,d. In Figure 10c, we used Λ = 1050 nm and h = 20 nm to make sure that only ESPMs were excited (the grating was too shallow to support cavity modes at the given spectral range). As expected for thin enough gratings, the resonant location was mainly determined by the materials and the grating period, not the groove width, as can be observed from Equation (3) (normal incidence). Therefore, we expected to observe two horizontal lines, as almost seen in Figure 10c, associated with the first (top line) and second (bottom line) ESPMs. In Figure 10d, Λ = 300 nm and h = 600 nm were used, and four CM branches were observed (in this case, Λ was too small to support ESPMs). Figure 10 clearly demonstrates the effect of the grating grooves on the resonant behavior of the reflecting grating, which has strong dependence on the grating period and thickness as shown above.

**Figure 11:**
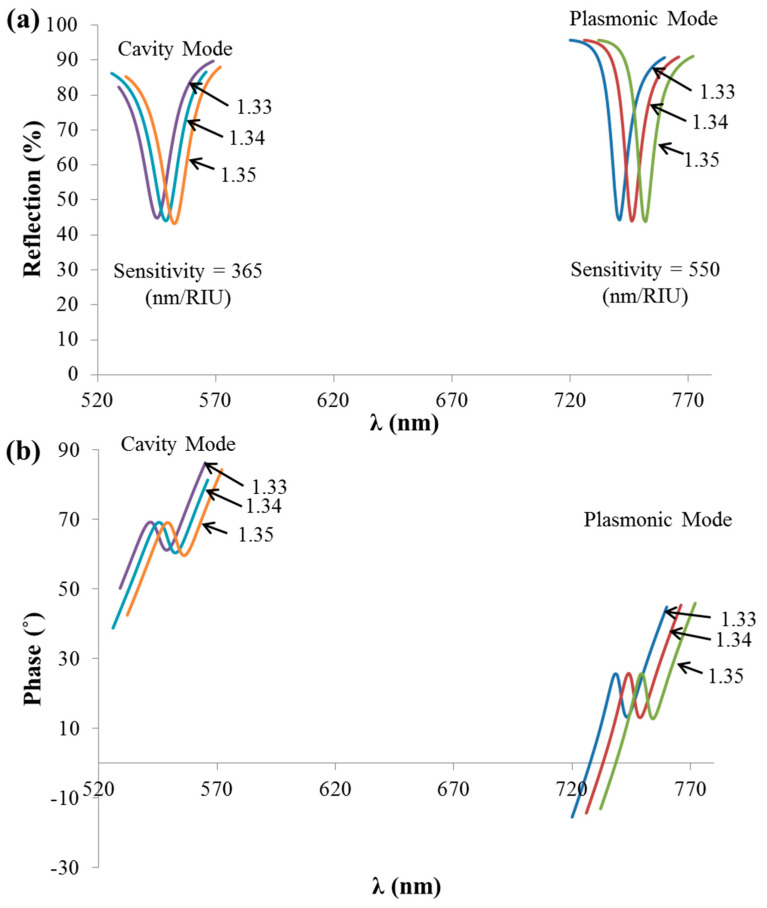
(**a**) Reflection spectra from the thick reflecting grating geometry at different analyte RIs. (**b**) Phase response at the same RIs as in Figure 11a. TM-polarized light under normal incidence was used in the simulations of the Ag grating FF = 0.85. Si was used for the substrate. For the CM calculations, Λ = 300 nm, h = 600 nm were used, while for the SPM calculations, Λ = 1050 nm, h = 20 nm were used.

Intensity versus phase detection using the reflecting grating is shown in Figure 11. Figure 11a shows the spectral reflection variation at different analyte RIs (1.33, 1.34, and 1.35), while Figure 11b shows the spectral phase response for the same RIs in Figure 11a. As can be observed in Figure 11b, the phase response was not sharp as in the case of the analyte mode phase response in Figure 7b. This is expected to be a result of the losses of the metal in the case of the plasmonic scheme (Figure 9), supporting the excitation of ESP and cavity modes. The spectral reflection sensitivities of the ESPM and CM were 550 and 365 (nm/RIU), respectively, as indicated in Figure 11a. The fact that the ESPM was more sensitive to the analyte RI than the CM is believed to be a result of the enhanced interaction of the EMF with the analyte in the case of the ESPM. In the case of the CM, the field was strongly confined within the narrow grooves of the grating, which reduced the interaction with the analyte material.

**Figure 12:**
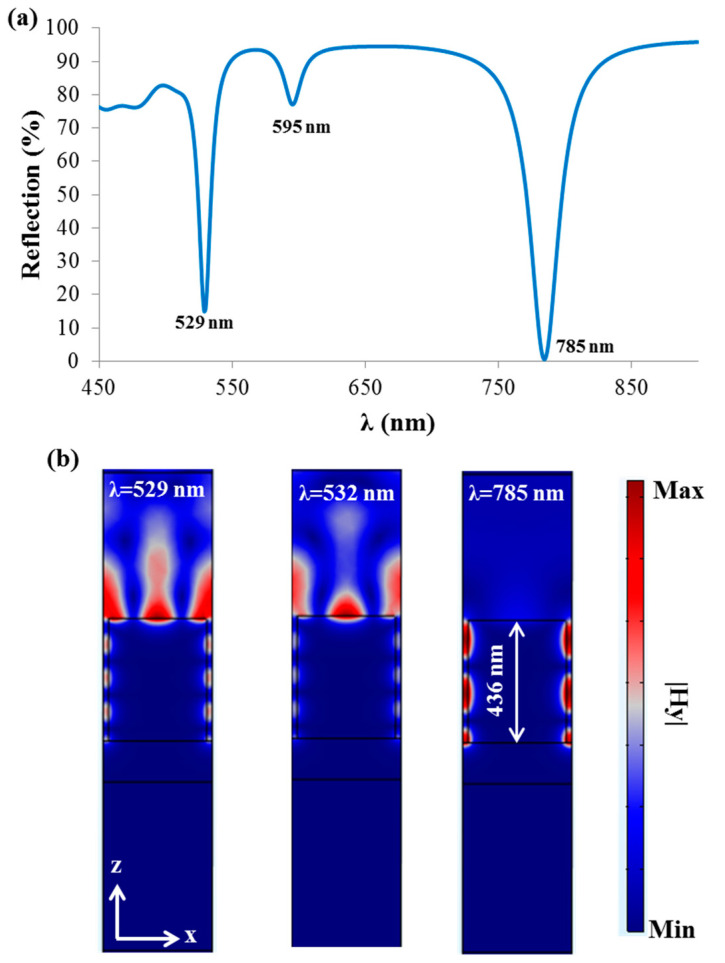
(**a**) Reflection spectra from the thick reflecting grating geometry (with Λ = 390 nm, h = 436 nm, and FF = 0.9) at 1.33 RI. (**b**) FD at the resonant wavelengths 529 nm and 785 nm as well as at 532 nm to simultaneously demonstrate the SEF and SERS enhancement. TM-polarized light under normal incidence was used in the simulations of the Ag grating. Si was used for the substrate.

To summarize this section, using a simple setup, one can measure ESP, CM resonance shift, and SEF and SERS signals, thus forming a multimodal sensing or imaging system [23]. To illustrate the concept of multimodal sensing geometry in Figure 9, Λ = 390 nm, h = 436 nm, and FF = 0.9 were chosen, and three resonances were observed at the spectral range 450–900 nm (529 nm, 595 nm, and 785 nm) in Figure 12a. At this point, our interest was to propose a geometry exhibiting resonances at 532 nm and 785 nm for SEF and SERS signals enhancement, respectively. Although the first resonance was observed at 529 nm, 532 nm was still included within the resonant spectral region. Figure 12b shows the FD at the resonant wavelengths 529 nm and 785 nm as well as at 532 nm to verify the field confinement on the grating surface (at 529 nm and 532 nm) and grooves (at 529 nm, 532 nm, and 785 nm). Besides this, all the resonant dips can be also used for RI sensing.

Multimodal systems are important to provide as much information as possible on the measured samples, such as the concentration of analytes and characterization of cells and tissue. Reflecting nanogratings (having nano-grooves) can easily exhibit multiple resonant modes (ESP and cavity modes) appearing simultaneously at several wavelengths, making the proposed nanograting geometry a potential nanostructure with a simple setup for multiple sensing operations, such as RI or yes/no sensing in the visible and infrared (IR) ranges and SERS and SEF sensing modes. Since this scheme was pure plasmonic geometry, the phase response was less sensitive than in the previous case of the TDGTMF in Figure 3. On the other hand, the ESPM sensitivity in the reflecting grating is comparable to the analyte mode sensitivity of the TDGTMF in which the CM was less sensitive.

### 2.3. Thick Subwavelength Dielectric Grating-Based Sensor

**Figure 13:**
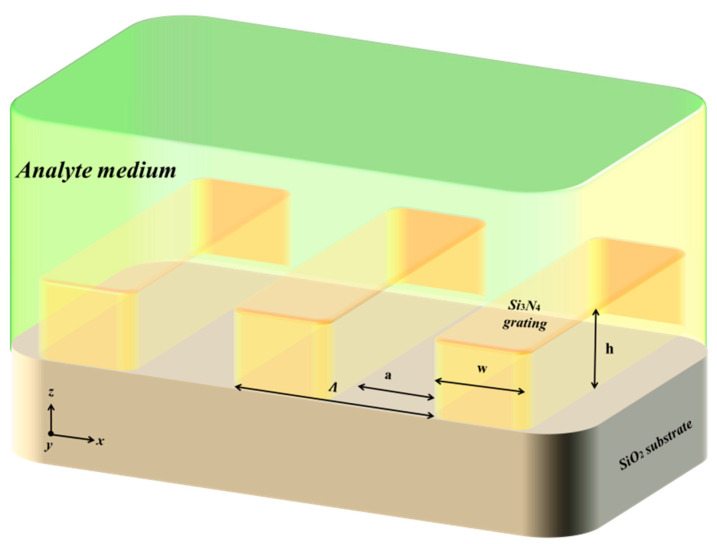
Schematic diagram of the thick SW dielectric grating-based sensor geometry.

The use of thick enough subwavelength dielectric grating (without planar waveguide layer) was demonstrated in the third grating configuration in which resonances above a certain grating thickness were observed. Experimental demonstration of the SWG as a chemical sensor in the short-wave IR spectral range was shown by the authors of [20]. In comparison to the conventional GMR structure with the waveguide layer, the thick SWG has several unique advantages: (i) Higher sensitivity is achieved when the grating spaces are filled with the analyte material peaking at certain space values as a result of the increase in the analyte-evanescent field interaction volume; (ii) the sensitivity increases with the grating thickness; (iii) a sudden increase in the quality (Q)-factor of the resonance is observed at a specific grating thickness h value accompanied by local field enhancement of the (~10^3^) characteristic of a nano-antenna-type pattern [20]. Figure 13 shows the schematic diagram of the thick SW dielectric grating-based sensor geometry without planar waveguide layer as used in the conventional GMR geometry.

**Figure 14:**
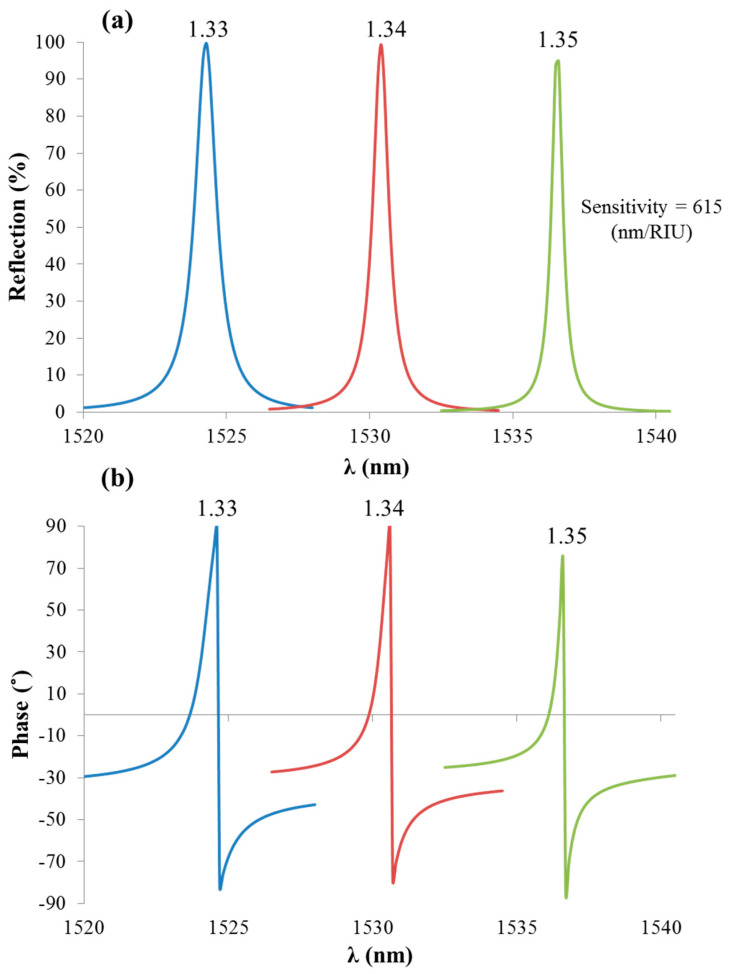
(**a**) Reflection spectra from the thick SW dielectric grating geometry at different analyte RIs. (**b**) Phase response at the same RIs as in Figure 14a. TE-polarized light under normal incidence was used in the simulations of the Si3N4 grating with Λ = 1000 nm, h = 1000 nm, and FF = 0.4. SiO_2_ was used for the substrate.

Figure 14a shows the spectral reflection variation at different analyte RIs (1.33, 1.34, and 1.35) of the geometry in Figure 13. In Figure 14a, the FWHM of the resonance was larger than that in Figure 14b and showed a sharper response when detecting the phase response than the same RIs in Figure 14a accumulated with enhancing the FOM. In comparison to the phase response of the analyte mode in the TDGTMF in Figure 7b, the phase response of the GMR in the thick dielectric grating (without waveguide layer) showed an even sharper response, with the phase jump close to π around the resonance. This is because the structure in Figure 13 was pure dielectric, while in the TDGTMF in Figure 3, an MF existed below the dielectric grating. The spectral reflection sensitivity of the GMR peak to the analyte RI was 615 (nm/RIU), as indicated in Figure 14a. Transverse-electric (TE)-polarized light under normal incidence was used in the simulations of the Si3N4 grating with Λ = 1000 nm, h = 1000 nm, and FF = 0.4. SiO_2_ was used for the substrate.

### 2.4. CD-R-Based off-the-Shelf Plasmonics

Experimental and theoretical investigation on the SPR sensing using CDs with different track pitches, including BD, DVD, and CD-R, were shown by the authors of [30]. In typical BDs, DVDs, and CDs, the standard track period is 320 nm, 740 nm, and 1600 nm, respectively [30]. The authors of [30] performed a treatment of the CD structures. In the study, the recording dye layer and reflective alloy layer of the BD were removed using a specific procedure. This procedure was followed by sputtering deposition of 2 nm chromium and 80 nm gold. On the other hand, the protective polycarbonate layer and the photosensitive dyes of DVD and CD-R were removed by following the procedures described by the authors of [31,32], and the exposed aluminum reflective layers were used directly in SPR sensing.

The authors of [33] proposed a simple angular displacement measurement system with sub-micro radian resolution based on SPR excitation in CDs. The authors presented an AFM test and schematic of the layers forming the CD in which the protective lacquer layer was removed from the CD to enable the attachment of biological entities for sensing application. Removing the protective layer also efficiently excited the plasmon’s at the air-metal interface (after removing the lacquer layer).

The preliminary results found by the authors of [34] showed SPR angle modification using a biomolecular layer on the CD-based biosensor structure. The authors of [35] presented an SPR-based CD biosensor with a circular fluidic channel. In the AFM test before 50 nm gold layer sputtering, the authors showed a grating groove depth of 64 nm and width of 468 nm. After sputtering, a groove depth of 54 nm and groove width of 445 nm were shown in the AFM test.

Simpler treatment procedure was performed by the authors of [36] to isolate the metal layer. First, the authors cut the DVD disc into slices by scissors. Then, they easily separated the glued metal layer and the organic dye layer, and they finally washed the side with the metal layer using isopropanol to remove the residual dye. AFM of the prepared metal grating showed a grating period of 740 nm and a peak-to-valley modulation depth of 86 nm [36]. The structure was used for RI sensing. Additional works on SPR based DVD structures can be found in [37,38,39,40,41]. Theoretical and experimental study of the SPR supported by CDs can be found in [42], biosensing application of different types of CDs is discussed in [43].

Many other applications can be implemented using CDs-based structures, for example, SERS-based CDs have been described by the authors of [32,44,45]. Moreover, schematic, SEM, and AFM characterization of the CDs-based structures and the materials used to manufacture the different types of CDs have been described by the authors of [30,32,33,34,35,36,37,38,39,40,41,42,43,44,45,46,47], including the treatment methods made on the sample to prepare them for sensing applications. The main treatment method is exposure to metallic film. Therefore, the sample can have direct contact with the analyte as much as possible to enhance the plasmonic response of the structure. Determination of the dye-recording layer thickness was shown by the authors of [48]. The authors of [49] transform a CD into a simple and cheap photocatalytic nanoreactor.

In the abovementioned works, many efforts were made on the optical recording discs to transform them into plasmonic substrates. In this experimental work, we have three main advantages. First, we proposed the use of the CD-R-based sensor with almost zero treatment. Second, we proposed measuring the TM response in relative to the TE one so the contrast of the measured reflection dips could be significantly improved. Finally, to the best of our knowledge, we implemented phase detection SPR-based sensing using off-the-shelf CDs for the first time.

**Figure 15:**
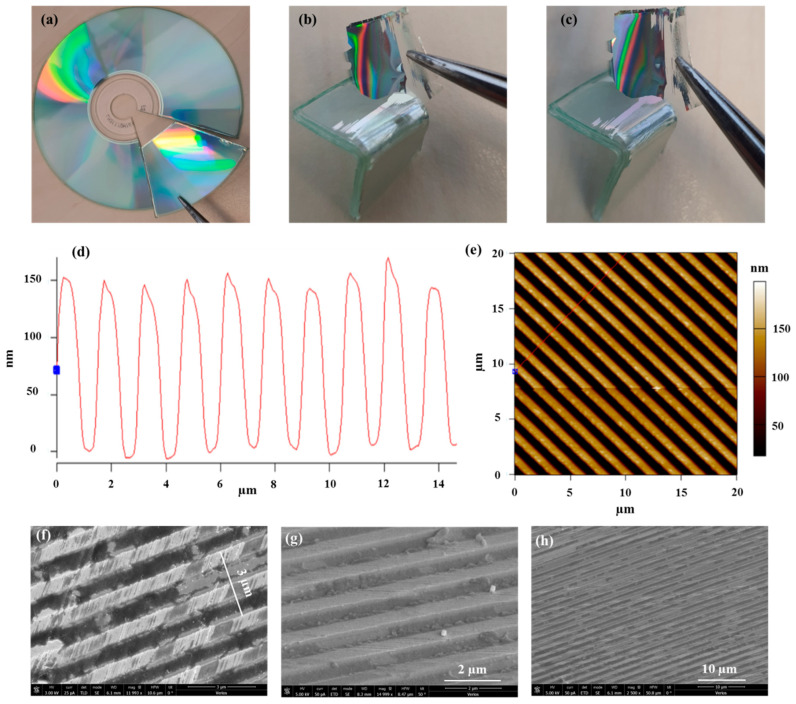
(**a**) The piece cutting from the CD. (**b**,**c**) The extracted sample at different angles. (**d**) AFM characterization of the prepared sample showing the grating height of ~150 nm. (**e**) AFM characterization of the prepared sample showing the grating period of ~1500 nm. (**f**–**h**) Different SEM characterization of the prepared sample showing the grating structure.

Figure 15 shows the characterization of a prepared sample in which almost zero treatment was performed when preparing the substrates. When preparing the substrate, we only separated the top part, including the marketing sign, from the bottom part. Figure 15a shows the cut piece from the CD. Figure 15b,c show the extracted sample at different angles, where the colors verify the existence of the grating. The clear advantage of using off -the -shelf CDs is first the almost zero treatment as well as the possibility of preparing the sample at the desired size from the CD area. The grating height profile is shown in Figure 15d with an estimated grating height of ~150 nm. Figure 15d was extracted from the AFM characterization of the prepared sample shown in Figure 15e, which also presents the grating period profile with an estimated periodicity of ~1500 nm. The AFM data and the different SEM characterization of the prepared sample (Figure 15f–h) clearly verify the existence of the grating structure. According to the XPS test, the scratches on the grating lines shown in Figure 15f are the remaining lacquer material used to connect the different parts of the CD-R.

**Figure 16:**
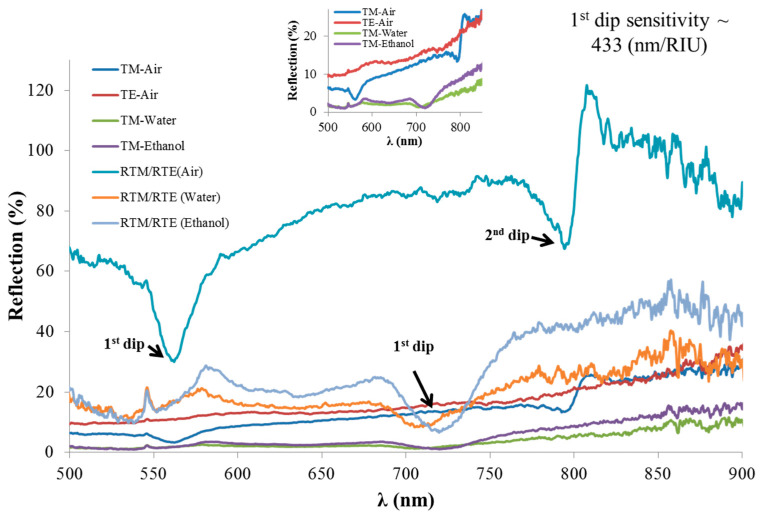
Experimental reflection spectra from the prepared sample extracted from the CD-R at different analytes in the visible range.

Figure 16 shows experimental reflection spectra from the prepared sample extracted from the CD-R at different analytes in the visible range. The TE reflection spectrum when air was used is shown in the red curve without any signature of resonant behavior. On the other hand, the TM reflection spectrum showed two dips in the blue curve, as can be clearly seen in the zoomed sub-figure in the top part of Figure 16. A shift of the left-side dip (called the first dip), observed when air was used (blue curve), occurred at the TM response measured at deionized water (DI) and ethanol analytes, as shown in the green and purple curves, respectively. The DI water and ethanol were simply introduced on the sample using a pipet and they covered the whole surface.

Important conclusions arising from the initial experiments are as follows: The resonances were only seen under TM-polarized light because the plasmonic excitation required this polarization. The sensitivity was determined by parameters of the structure, the materials, and the wavelength. Usually, the higher the interaction between the evanescent field and the analyte, the higher the sensitivity [1], and this can be achieved by specific design. Here, as a metallic grating without extra layers, the sensitivity was expected to be high enough. Second, the contrast of the resonances was relatively low, and similar results have been reported in many other works on sensing using CD-R structures. Therefore, the first improvement we propose is that almost zero treatment is required to prepare the substrate. Additionally, we suggest measuring the TM response relative to the TE one. We also suggest mirroring the response (as the reflective sample), as significant improvement was observed in the contrast of the resonances. The results of the reflection spectra when normalizing the TM reflection spectrum to the TE one (RTM/RTE) are shown in the azure, orange, and gray curve for air, water, and ethanol analytes, respectively. The fact that RTM/RTE exceeded 100% reflection when air was used is because, near the second reflection dip and at the spectral range of 803–823 nm, the TM reflection was larger than the TE one. The spectral sensitivity of the first reflection dip was ~ 433 (nm/RIU) as indicated in Figure 16.

It was important and necessary to verify the reliability of the results and that they can be easily reproduced at a high precision level. We performed reflection measurements from tens of prepared samples, and the reproduced measurements achieved always provided comparable results. This means that the resonances were observed at similar spectral position with only few nanometers shift between the different samples. In addition, a very comparable contrast of the reflection dips was observed for the different samples.

**Figure 17:**
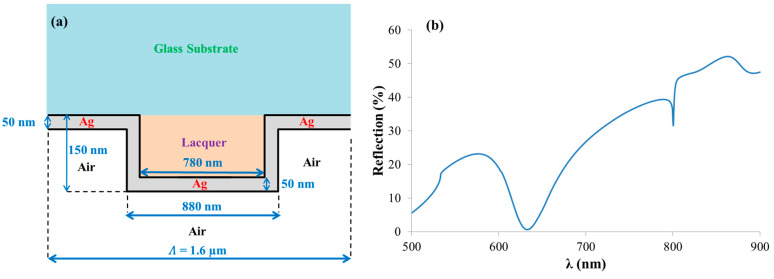
(**a**) Schematic diagram of the structure used in the reflection spectra simulation shown in (**b**). TM-polarized light and normal incidence were used in the simulation.

The schematic diagram of the prepared sample is shown in Figure 17a. The geometrical parameters used in the simulation are written in the schematic diagram, where the grating profile parameters are close to those shown in the SEM and AFM data in Figure 15. TM-polarized light and normal incidence were used in the reflection simulation and are shown in Figure 17b. The simulation showed good agreement with the experimental results in Figure 16 (blue curve) when comparing the right-hand reflection dip spectral position, contrast, and shape. On the other hand, the spectral position of the left-hand reflection dip in the simulation was different from the one observed in the experiment. This may be due to several reasons. First, the difference between the parameters used in the simulation and the real ones may have affected the position, as determining the real values of the different layers thicknesses is not an easy task. Second, the remained lacquer on the metallic grating can affect the reflectance. Figure 17 is only a qualitative attempt to estimate the reflection of the structure. The most important component is that the experimental results are reproducible as discussed below.

Next, to the best of our knowledge, we present phase detection using the CD-R-based grating for the first time. Phase measurements can significantly improve the FOM as shown in the numerical simulations for the three different configurations above, except for the thick metallic gratings case.

**Figure 18:**
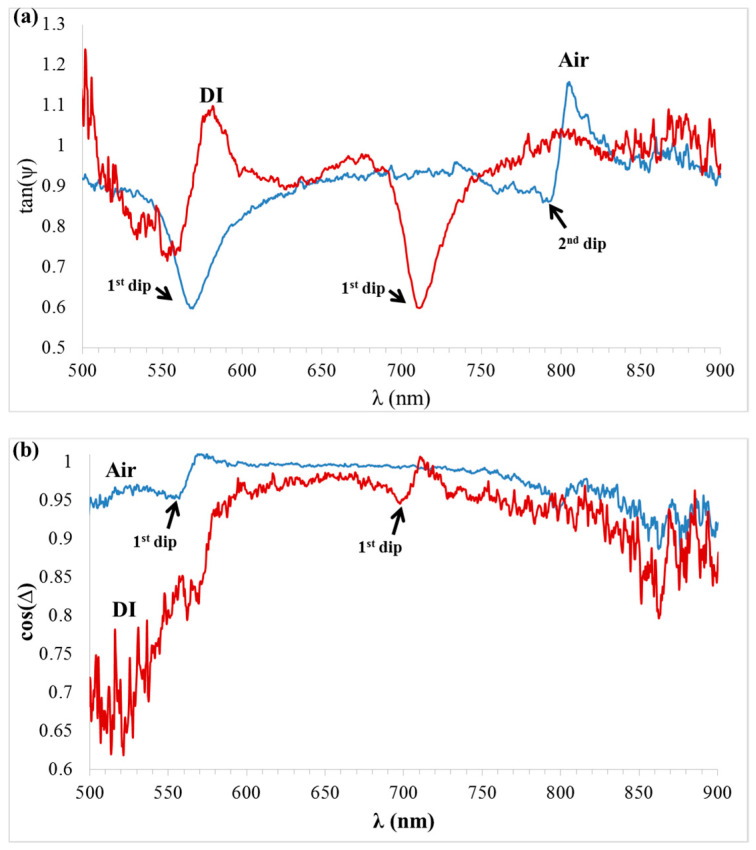
Demonstration of phase detection using CD-R and polarimetric functions (**a**) tan(ψ) and (**b**) cos(Δ) response for air and DI analytes under TM polarization.

Demonstration of phase detection using CD-R is shown in Figure 18, where the polarimetric functions tan(ψ) and cos(Δ) were extracted using the equations and experimental setup as shown by the authors of [28,50,51]. Briefly, the CD sample was inserted between two polarizers, while the optics axis (perpendicular to the grating lines) was oriented at 45° with respect to the first polarizer. The ellipsometric parameters tan(ψ) and cos(Δ) can be extracted by measuring the light intensity at three points based on three analyzer rotation angels—0°, 45°, and 90°—relative to the first polarizer [28,50,51]. Figure 18 shows initial results to demonstrate the phase detection potential of the CD-R-based grating structure. The response for air and DI analytes are shown in the blue and red curves, respectively, under TM polarization.

**Figure 19:**
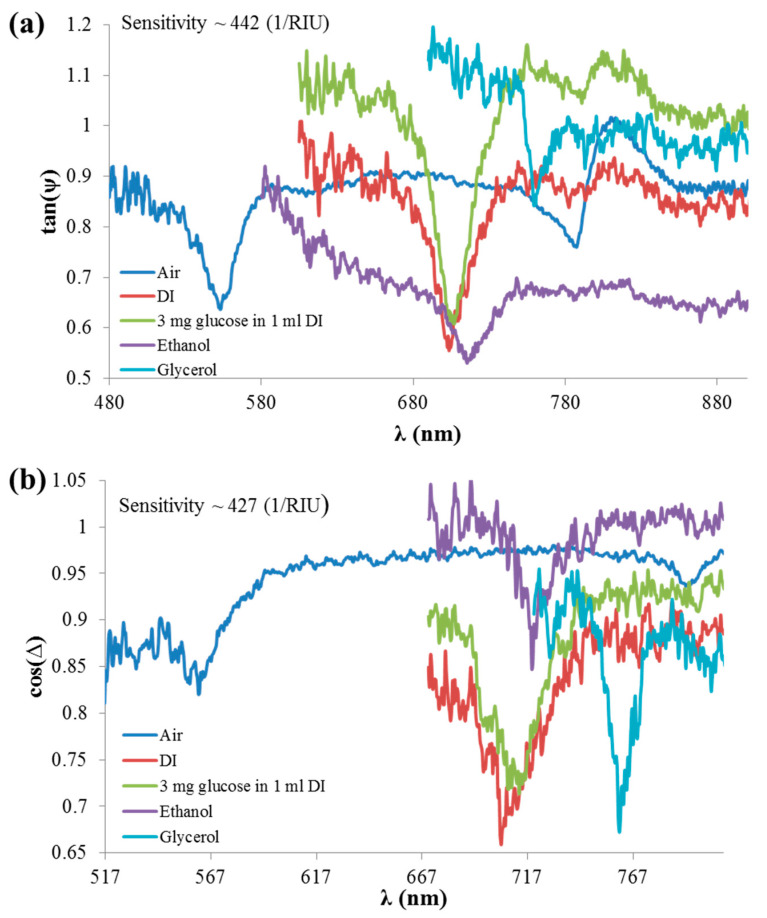
Demonstration of sensing operation-based phase detection using CD-R and polarimetric functions (**a**) tan(ψ) and (**b**) cos(Δ) response at different analytes.

To verify the reproducibility of the results, additional measurements of the polarimetric functions using different sample are presented in Figure 19. It should be noted that, because the phase measurements were much more sensitive than intensity measurements, some noise was observed in the phase measurements. We plan to improve this, as the reported results are only initial results aimed to demonstrate the phase detection method in the CD-R-based gratings for sensing applications.

Figure 19a,b show the spectral response of tan(ψ) and cos(Δ,) respectively, for five types of analytes: Air, DI, 3 mL glucose in 1 mL DI (3% wt.), ethanol, and glycerol. The sensitivity of the first dip was detected, and a sensitivity of 442 (1/RIU) and 427 (1/RIU) was measured for tan(ψ) and cos(Δ) as indicated in Figs 19a and 19b, respectively. The sensitivity of tan(ψ) and cos(Δ) was calculated by extracting the resonant wavelengths in Figure 19 as a function of wavelength and then calculating the slope of the curve.

**Figure 20:**
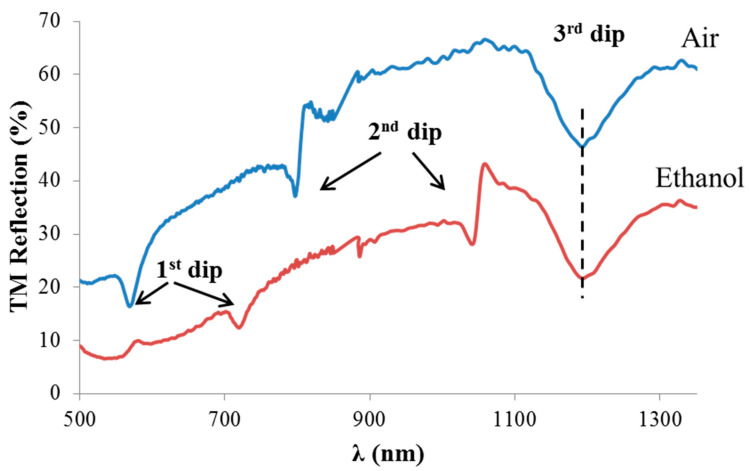
Observation of resonant dips in the IR spectral range showing the potential of sensing operation in the IR using CD-R.

The potential of sensing operation in the IR using CD-R is demonstrated in Figure 20 in which new prepared CD-R sample was used. A spectral shift of the first and second dips was observed when changing the analyte from air to ethanol. On the other hand, the third dip position almost remained fixed, which might indicate the possibility of self-referenced sensing operation using CD-R. The results in Figure 20 are only initial results, and more experiments are required for demonstration sensing in the IR range.

Table 1 summarizes the parameters and unique features of all the nano-scale grating configurations mentioned above. In addition, the data regarding the thin metallic nanoslits on dielectric substrate for enhanced optical transmission resonance excitation is also included and compared to the other grating-based sensing geometries.

## 3. Conclusions

The effect of nano-scale features of resonant metallic and dielectric-based sensing configurations is discussed in three different configurations: Thin dielectric grating on thin metal film, reflecting grating, and thick dielectric grating. Among the unique features of the proposed SWG/nanograting structures, we highlight the following: (a) The flexibility to design the sensors for sensing small (molecules, viruses) and large (cells) entities utilizing the excited LRSPR; (b) the flexibility of sensing different types of analytes; (c) the possibility of working at several operation modes: Spectral, angular, intensity, phase detection; (d) the flexibility of tuning the operation spectral range (visible, IR) by tuning the nano-scale features of the grating parameters; (e) planar and compact configurations, which are preferable over prism-based structures in the case of miniaturization and implantation in integrated photonics and plasmonics.

Comparable spectral sensitivity was achieved for the GMR in metal mode excited in the TDGTMF geometry, the ESPM excited in the reflecting grating, and the GMR excited in the thick subwavelength dielectric grating, while the CM excited in the reflecting grating showed smaller sensitivity. On the other hand, the thick subwavelength dielectric grating showed the sharper phase response at resonance, while in the other cases, the phase sharpness decreases due to the loss of metal. Self-referenced operation was achieved in the TDGTMF geometry, which makes the measurement stable and less sensitive to temperature fluctuations and optomechanical drifts. Multimodal sensing operation can be easily achieved using the reflecting grating by measuring ESP, CM resonance shift, and SEF and SERS signals by designing the grating parameters to have the resonances at the desired wavelengths. Multimodal systems are important to provide as much information as possible on the measured samples, such as the concentration of analytes and characterization of cells and tissue. Higher spectral sensitivity and sharper phase response were achieved in the pure dielectric configuration, which showed of thick subwavelength dielectric grating on the dielectric substrate.

Improved intensity or wavelength and phase detection methods were achieved from off-the-shelf CD-R devices with almost zero treatment and with comparable spectral sensitivity in comparison to the configurations mentioned. These improved methods offer the advantages of low cost, availability, and large sensing area.

## Figures and Tables

**Table 1:** Summary of the parameters and unique features of all the grating-based geometries.

Geometry	Thin Dielectric Grating on Thin Metal Film (TDGTMF)	Reflecting Grating	Thick Dielectric Subwavelength Grating	Enhanced Optical Transmission (EOT) Grating	Off-the-Shelf Compact Disc-Recordable (CD-R)-Based Grating
Spectral Operation range	SWIR	Visible	SWIR	Visible, SWIR	Visible, NIR
Grating period (nm)					
Grating thickness (nm)	1000	1050	1000	450, 1120	1500
Spectral Sensitivity (nm/RIU)					
	175	20, 600	1000	45, 20	150
Signature in reflection spectrum					
Unique feature	580 (GMR on metal)	550 (Plasmonic Mode), 365 (Cavity Mode)	615 (GMR)	435 [29], ~1100 [13]	~430–440
		Dip			
	Dip			Dip	
			Peak		Dip
Preparation procedure					
		Multimodal sensing operation mode			
	Self-referenced sensing operation mode			Self-referenced sensing operation mode	
			(a) Sharp phase response		(a) Low cost
			(b) Enhanced field at certain grating thickness		(b) Widely available
					(c) Large sensing area
		Lithography,	Lithography,	Lithography,	
	Lithography,	lift-off processes	lift-off processes	lift-off processes	
	lift-off processes				
					Off-the-shelf

## Data Availability

The data that support the findings of this study are available from the corresponding authors upon reasonable request.
